# Advancing veterinary vaccines design through trained immunity insights

**DOI:** 10.3389/fvets.2024.1524668

**Published:** 2025-01-15

**Authors:** Xin Wang, Guohua Yu

**Affiliations:** ^1^College of Life Science, Longyan University, Longyan, China; ^2^Fujian Provincial Key Laboratory for the Prevention and Control of Animal Infectious Diseases and Biotechnolog, Longyan, China; ^3^Key Laboratory of Preventive Veterinary Medicine and Biotechnology, Longyan University, Longyan, China; ^4^Chinese International College, Dhurakij Pundit University, Bangkok, Thailand

**Keywords:** trained immunity, innate immune memory, metabolic reprogramming, epigenetic modification, veterinary vaccines

## Abstract

Trained immunity, characterized by long-term functional reprogramming of innate immune cells, offers promising new directions for veterinary vaccine development. This perspective examines how trained immunity can be integrated into veterinary vaccine design through metabolic reprogramming and epigenetic modifications. We analyze key molecular mechanisms, including the shift to aerobic glycolysis and sustained epigenetic changes, that enable enhanced immune responses. Strategic approaches for vaccine optimization are proposed, focusing on selecting effective trained immunity inducers, developing innovative adjuvant systems, and achieving synergistic enhancement of immune responses. While implementation challenges exist, including individual response variations and safety considerations, trained immunity-based vaccines show potential for providing broader protection against emerging pathogens. This approach could revolutionize veterinary vaccinology by offering enhanced efficacy and cross-protection against heterologous infections, particularly valuable for zoonotic disease control.

## 1 Introduction

In the field of veterinary medicine, vaccine design faces unprecedented challenges as rapidly mutating viruses, like Avian Influenza H5N1 and H7N9, undermine traditional vaccine efficacy. High mutation rates in these strains increase zoonotic spillovers and pandemic risks. H5N1’s genomic reassortment and expanded mammalian host range heighten human infection risks, while H7N9 mutations further reduce vaccine effectiveness (1, 2). These challenges underscore the urgent need for innovative vaccine strategies. Netea et al. (3) first proposed the emerging concept of “trained immunity,” suggesting that the long-term functional reprogramming of innate immune cells can significantly enhance the host’s defense against various pathogens. Further research by Netea et al. (4) demonstrated that trained immunity confers broad protection against heterologous pathogens through metabolic and epigenetic reprogramming. According to Netea et al. (5), whole-microbe-based vaccines can enhance innate immune responses by inducing trained immunity, thereby reducing susceptibility and disease severity against emerging pathogens such as SARS-CoV-2, providing new insights for vaccine design. This article explores the potential application of trained immunity in the design of veterinary vaccines and proposes new strategies to optimize vaccine development through metabolic reprogramming and epigenetic regulation, with the aim of improving vaccine efficacy and safety.

Trained immunity, through the long-term functional reprogramming of innate immune cells, has emerged as a cutting-edge paradigm in vaccine design, offering new strategies to address complex pathogens (6). Recent studies have further revealed that trained immunity not only enhances innate immune responses but also promotes adaptive immune functions, thereby providing broad protection against a variety of heterologous pathogens (7). This reprogramming process involves unique tolerance, activation, and differentiation mechanisms, enabling the immune system to respond more robustly to recurrent or novel pathogens. During the COVID-19 pandemic, research on trained immunity has underscored its critical role in antiviral defense and vaccine efficacy, particularly through mechanisms involving epigenetic modifications that amplify immune responses, offering fresh insights and theoretical support for the development of novel vaccines (8).

Kaufmann et al. (9) found that the BCG vaccine exhibits a significant protective effect against influenza viruses, though its protective capacity against SARS-CoV-2 remains limited. This finding highlights the impact of pathogen-specific pathologies and tissue tropism on the effectiveness of trained immunity. Against this backdrop, this article proposes innovative strategies to integrate trained immunity into veterinary vaccines design. Approaches to vaccine design based on trained immunity include optimizing inducers, developing novel adjuvant systems, and enhancing the spatial and temporal control of immune responses. Such integrated strategies contribute to increased vaccine efficacy, expanded protective spectra, and improved immune memory.

Moreover, recent advancements in vaccine delivery systems and production technologies are steadily advancing trained immunity-based vaccines toward practical application (10, 11). Nonetheless, the implementation of trained immunity faces several challenges, including individual variations in immune response, the need for a comprehensive safety assessment framework, and the standardization of efficacy evaluation metrics (12, 13). Future research should focus on elucidating the molecular mechanisms underlying trained immunity, developing more efficient inducers, and optimizing vaccine delivery strategies. Integrating trained immunity into the design of veterinary vaccines holds promise not only for enhancing vaccine efficacy but also for providing more robust protection against emerging viral threats.

## 2 Metabolic and epigenetic basis for vaccine innovation

**Figure 1:**
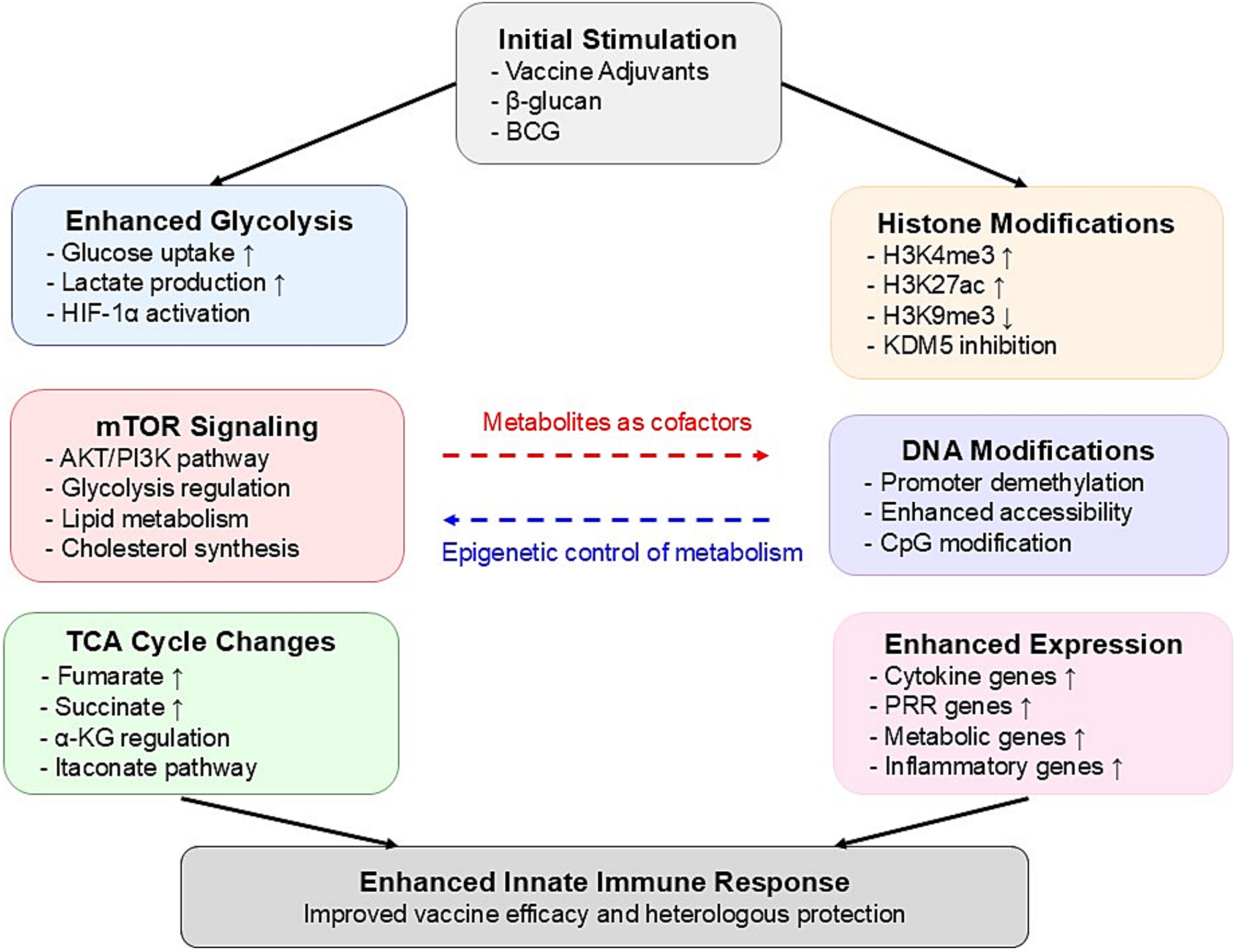
Mechanisms of metabolic and epigenetic reprogramming in trained immunity.

The molecular mechanisms underlying trained immunity-based vaccine innovation primarily involve two interconnected processes: metabolic reprogramming and epigenetic modifications. Figure 1 provides a comprehensive illustration of how metabolic reprogramming and epigenetic modifications are integrated in trained immunity, demonstrating the complex molecular networks that form the foundation for innovative vaccine design strategies. Understanding these processes is essential for developing more effective vaccines that can fully harness the potential of trained immunity.

### 2.1 Metabolic reprogramming in vaccine immunity

In trained immunity, metabolic reprogramming plays a critical role in determining the function and persistence of immune cells. Arts et al. (14) found that trained immunity drives a metabolic shift in innate immune cells, particularly monocytes and macrophages, from oxidative phosphorylation to aerobic glycolysis. This reprogramming, termed the “Warburg effect,” not only provides the energy and metabolic intermediates required to enhance immune responses but also establishes a link between metabolism and epigenetic regulation, with intermediates like fumarate stabilizing epigenetic marks critical for immune memory. Recent studies further highlight the relevance of this metabolic-epigenetic interplay in vaccine responses. For instance, Apps et al. (15) demonstrated that H5N1 vaccination induces persistent transcriptional changes in classical monocytes and CD8+ T cells, effectively modulating baseline immune states to enhance future responses. Similarly, Song et al. (16) revealed that mucosal vaccines not only elicit adaptive immunity but also induce durable trained immunity in myeloid cells through metabolic and epigenetic reprogramming, providing broad protection. These findings underscore the central role of metabolic reprogramming in trained immunity and suggest innovative directions for vaccine design to optimize long-lasting and broad-spectrum immune responses.

The metabolic regulation of trained immunity relies on key pathways within glycolysis and the tricarboxylic acid (TCA) cycle. Moorlag et al. (17) demonstrated that inducers like *β*-glucan enhance trained immunity through specific epigenetic modifications, and upon re-exposure to *Mycobacterium tuberculosis*, the induced monocytes produce heightened levels of pro-inflammatory cytokines, effectively limiting pathogen growth. This enhanced response is mediated by the AKT–mTOR-HIF1α signaling pathway, which serves as a core regulatory mechanism of trained immunity. Additionally, research by Bekkering et al. (18) found that the intermediate mevalonate, a product of the mevalonate pathway, promotes trained immunity via the IGF1R and mTOR signaling pathways. This process not only increases glycolytic activity but also promotes lipid metabolism and cholesterol synthesis, reinforcing the trained state of immune cells. The accumulation of mevalonate establishes a positive feedback loop that continuously enhances the effects of trained immunity. These studies illustrate how metabolic reprogramming functions in the induction and maintenance of trained immunity, offering potential new strategies for vaccine development.

### 2.2 Epigenetic modifications as Long-term mediators

The persistence of trained immunity relies heavily on epigenetic reprogramming, which induces long-term changes in gene expression without altering the DNA sequence. This reprogramming involves specific histone modifications and DNA methylation patterns that sustain the effects of trained immunity even after the initial stimulus has faded. Fanucchi et al. (19) proposed that a core mechanism of trained immunity is the interaction between epigenetic and metabolic pathways, which ensures that immune cells can respond rapidly to subsequent infections.

In terms of histone modifications, increased activating marks such as H3K4me3 and H3K27ac, along with the reduction of repressive marks like H3K9me3, are crucial for maintaining the trained immunity phenotype (20, 21). These modifications create an open chromatin state in the promoter regions of inflammatory genes, enabling faster activation of these genes upon secondary stimulation (22). Noz et al. (23) demonstrated in their study on the BCG vaccine that these histone modifications can persist in immune cells for several months, providing a molecular foundation for the long-term effects of trained immunity.

The process of trained immunity is accompanied by significant alterations in DNA methylation patterns, particularly in the promoter regions of immune-related genes (20). Genome-wide analyses have revealed specific methylation signatures associated with trained immunity, which correlate with an enhanced transcriptional response (21). These epigenetic modifications serve as potential biomarkers for successful induction of trained immunity and are closely linked to the metabolic reprogramming of innate immune cells (14). Notably, these DNA methylation changes persist even after the initial stimulus has been removed, suggesting their role in maintaining the trained immunity phenotype (20).

The interplay between cellular metabolism and epigenetic modifications forms a self-sustaining feedback loop that is critical for the long-term maintenance of the trained immunity phenotype (22). Key metabolic intermediates generated through metabolic reprogramming, such as acetyl-CoA, *α*-ketoglutarate, and NAD+, function as essential cofactors for epigenetic regulatory enzymes, bridging the metabolic network and epigenetic regulation within the cell (4). Activation of this metabolic-epigenetic axis promotes histone modifications and chromatin remodeling, thereby sustaining an enhanced transcriptional program and immune response (14). A deeper understanding of this metabolic-epigenetic regulatory network provides new therapeutic targets for optimizing vaccine design and immunotherapeutic strategies, with the potential to enhance immune memory formation and maintenance through targeted manipulation of metabolic pathways.

## 3 Strategic approaches for vaccine enhancement

**Figure 2:**
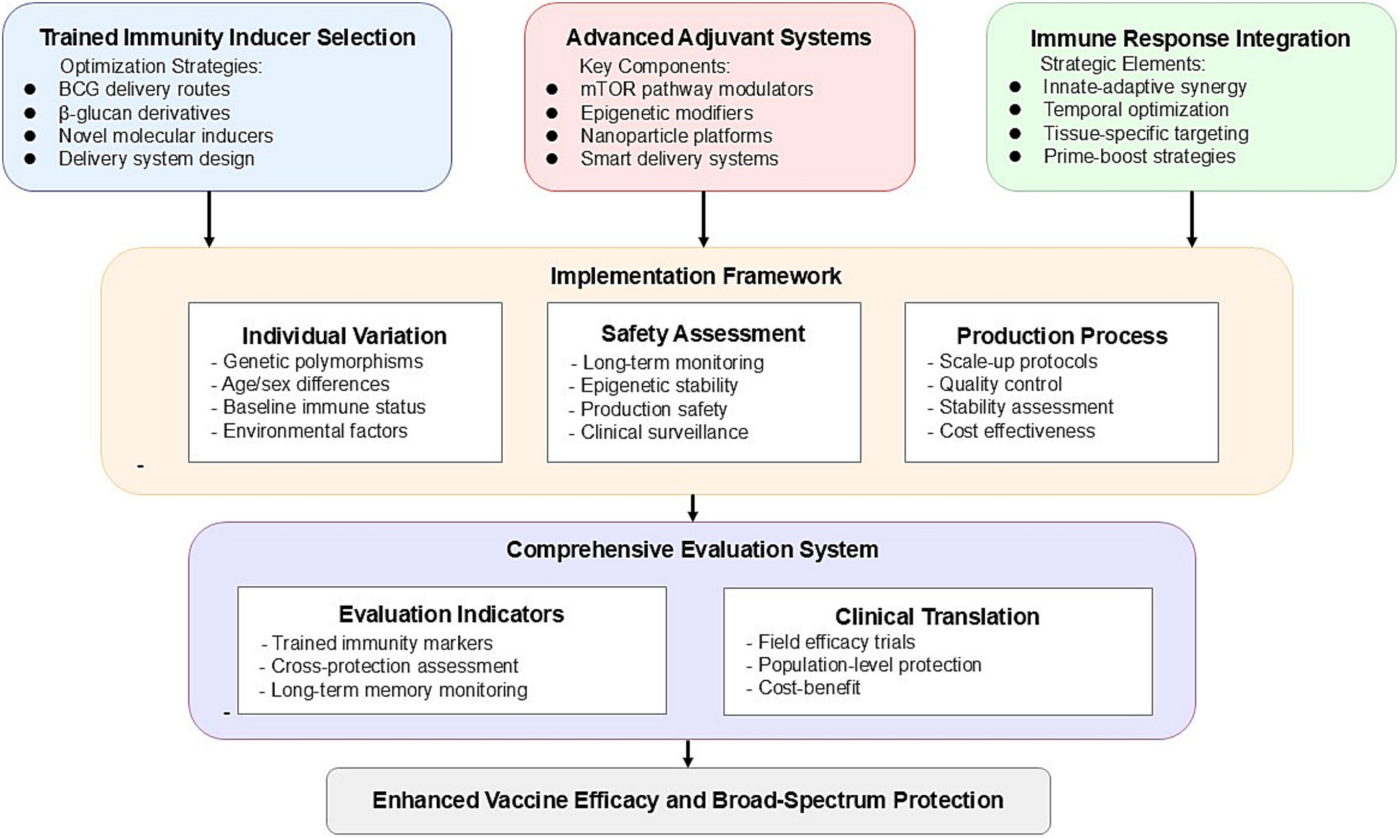
Roadmap for vaccine optimization based on trained immunity.

Integrating trained immunity into vaccine design requires a strategic approach across multiple dimensions. Here, we propose three key strategies that can significantly advance vaccine development by leveraging the mechanisms of trained immunity. Figure 2 presents a systematic roadmap for vaccine optimization based on trained immunity, integrating the key strategic approaches of inducer selection, adjuvant system design, and immune response enhancement into a coherent framework for future vaccine development.

### 3.1 Selection strategy for trained immunity inducers

Traditional inducers like BCG have provided a valuable foundation for the development of trained immunity-based vaccines. Netea et al. (7) noted that BCG can trigger long-lasting protection against a range of heterologous infections by reprogramming the epigenetic landscape of innate immune cells. Further research by Darrah et al. (24) demonstrated that intravenous administration of BCG offers significantly better protection against *Mycobacterium tuberculosis* (Mtb) in non-human primates compared to subcutaneous injection, underscoring the critical role of administration routes in the induction of trained immunity. This finding suggests that the optimal delivery method should be carefully considered in the development of new trained immunity-based vaccines to maximize the immune potential of inducers.

In addition, emerging molecular inducers, particularly *β*-glucan, show great promise as alternative candidates for the next generation of trained immunity vaccines. Domínguez-Andrés et al. (25) found that β-glucan induces trained immunity through a Dectin-1/Raf1-dependent pathway, offering a more defined molecular mechanism and greater control over immune responses. This inducer not only strengthens innate immune responses via metabolic and epigenetic reprogramming but also enhances adaptive immunity, achieving broader protection. Further research by Li et al. (26) highlighted that synthetic *β*-glucan variants can be designed for improved stability and targeted delivery, offering better safety and fewer side effects compared to traditional live-attenuated vaccines.

### 3.2 Innovative adjuvant systems design

The development of adjuvants oriented toward trained immunity represents a paradigm shift in vaccine design. Traditional adjuvants primarily focus on enhancing adaptive immune responses, while new designs should aim to optimize both innate and adaptive immunity (27). Recent advances in understanding the mechanisms of trained immunity have identified potential targets for adjuvant development, including mTOR pathway regulators and epigenetic modifiers (19).

For the delivery of trained immunity inducers, novel nanoparticle platforms have shown significant advantages. Li et al. (28) proposed that non-viral nanoparticle platforms can precisely control the temporal and spatial distribution of immune stimulants, effectively enhancing the stability and potency of trained immunity induction while minimizing systemic side effects. Further, Priem et al. (29) validated a bone marrow-targeting nanobiology platform that specifically induces trained immunity in myeloid progenitor cells within the bone marrow, demonstrating sustained trained immunity and significant antitumor effects in a mouse melanoma model. This MTP10-HDL nanotherapy reprograms multipotent progenitor cells epigenetically, promoting myeloid cell generation and overcoming immune suppression within the tumor microenvironment, thereby enhancing the efficacy of checkpoint inhibition therapy.

### 3.3 Synergistic enhancement of multiple immune responses

Combining innate and adaptive immunity has become a crucial direction for enhancing vaccine efficacy. While traditional vaccines primarily focus on adaptive immunity, growing evidence suggests that trained immunity can bolster both arms of the immune system. O’Neill and Netea (30) noted that the BCG vaccine not only enhances innate immune responses but may also reduce susceptibility to SARS-CoV-2 infection during the COVID-19 pandemic, demonstrating the non-specific protective potential and synergistic effects of trained immunity.

The timing of vaccination plays an important role in determining the strength and duration of the immune response. The timing of trained immunity induction relative to pathogen exposure or subsequent vaccinations can significantly impact protective efficacy. Yao et al. (31) showed that early induction of trained immunity can enhance the anti-infective response upon later exposure, supporting a prime-boost strategy that incorporates trained immunity inducers to provide prolonged protection.

Spatial targeting has emerged as an effective strategy for controlling the distribution of trained immunity effects. Chavakis et al. (32) highlighted that trained immunity can be concentrated in specific tissues, such as tissue-resident macrophages, through targeted delivery systems, thereby avoiding systemic side effects. Localized trained immunity is particularly valuable for tissue-specific protection; for instance, in lung infection control, tissue-resident immune cells can maintain the trained immunity phenotype independently of circulating cells, offering new opportunities for tissue-specific vaccine strategies. Divangahi et al. (6) further clarified the relationship between trained immunity and other immune processes, providing a theoretical basis for establishing rational standards for tissue-specific vaccine development.

## 4 Implementation challenges and solutions

While trained immunity-based animal vaccine strategies show great promise, several key challenges remain in their implementation. Understanding and overcoming these challenges is essential for translating theoretical advances into practical animal vaccines.

The primary challenge is the significant variability in trained immunity responses among individual animals. Cirovic et al. (33) demonstrated that BCG vaccine-induced trained immunity leads to sustained transcriptional program changes at the level of hematopoietic stem and progenitor cells, but this response varies markedly between individuals. Such heterogeneity may be influenced by HNF1 gene polymorphisms, age, sex, and environmental factors. Additionally, baseline cytokine production capacity and chromatin accessibility of immune-related genes prior to vaccination can predict trained responses (34). In livestock and poultry, trained immunity has demonstrated variability not only between individuals but also across species and breeds (35). For instance, *β*-glucan supplementation in chickens has been shown to enhance immune response differentially between broiler and layer breeds, with varying impacts on nitric oxide production and CD40 expression (36). Similarly, in cattle, aerosolized BCG vaccination induces a robust trained immunity phenotype in monocytes, enhancing cytokine production and resistance to subsequent challenges (37). Such findings underscore the importance of developing predictive models tailored to specific breeds or species. By assessing baseline immune markers and genetic predispositions, vaccination strategies can be optimized to account for breed-specific responses. Additionally, the customization of formulations for different animal populations promises to improve the efficiency of trained immunity induction and its application in disease management across diverse agricultural systems.

Secondly, the safety assessment framework for animal vaccines must account for the unique aspects of trained immunity. Given that trained immunity involves epigenetic modifications and metabolic reprogramming, a long-term safety monitoring system is essential. This framework should include immediate inflammatory response monitoring, tracking of long-term epigenetic changes, and safety evaluations under different physiological conditions (e.g., pregnancy, growth stages). For production animals, it is also necessary to assess the potential impacts of trained immunity on growth performance and product quality.

In terms of production, integrating trained immunity inducers into existing animal vaccine manufacturing processes presents technical challenges. Key issues include maintaining the stability of active components, ensuring batch consistency, and establishing standardized processes suitable for large-scale production. When developing novel delivery systems, special consideration should be given to the ease and cost-effectiveness of administration in animals. A modular production strategy is recommended, where formulation design and process parameters are optimized to ensure the stability and activity of trained immunity inducers.

Finally, current animal vaccine evaluation standards need to be expanded to comprehensively assess the protective effects of trained immunity-based vaccines. This requires establishing a multidimensional evaluation framework that not only develops reliable quantitative metrics for assessing the induction of trained immunity but also devises scientific methods to evaluate cross-protection against heterologous pathogens. The evaluation framework should assess protective effects at the population level, focusing on the vaccine’s effectiveness in real-world production environments. Additionally, due to differences in immune characteristics across animal species, the evaluation system should establish long-term monitoring schemes for immune memory persistence tailored to different species, ensuring durable protection across various animal types. This comprehensive evaluation framework will provide a more reliable scientific basis for the development of trained immunity-based animal vaccines.

## 5 Discussion

Integrating trained immunity into the design of veterinary vaccines represents a significant innovation in veterinary vaccinology. This strategy not only provides broad-spectrum pathogen protection but also offers new approaches to address key challenges faced by current animal vaccines. Traditional animal vaccines typically rely on specific immune responses, whereas the incorporation of trained immunity opens a pathway to enhance innate immunity, offering a novel strategy to improve overall vaccine efficacy.

Integrating trained immunity into veterinary vaccines represents a transformative strategy to enhance efficacy and address challenges associated with zoonotic diseases. The success of the BCG vaccine, which induces broad protection through metabolic and epigenetic reprogramming, provides a critical reference point. Studies have demonstrated BCG’s trained immunity effects against *Mycobacterium tuberculosis* (24) and potential cross-protection against SARS-CoV-2 (30), as well as its effectiveness against influenza viruses (9), suggesting its application for controlling diseases like avian influenza in poultry. These insights highlight how trained immunity principles can enhance innate immune responses, providing cross-protection and durable immune memory. By leveraging such strategies, veterinary vaccinology can better address rapidly mutating pathogens and zoonotic risks, contributing to animal health and advancing the One Health initiative.

Trained immunity-based animal vaccine design holds multiple implications. Firstly, by leveraging mechanisms of metabolic reprogramming and epigenetic regulation, trained immunity can activate and strengthen the innate immune system, thereby increasing cross-protection against emerging and mutated viruses. Dagenais et al. (38) noted that trained immunity provides effective defense against various pathogens through non-specific immune memory, which is particularly valuable in animal vaccine development as it aids in broader infectious disease control. Secondly, the synergy between trained and adaptive immunity could lead to longer-lasting protection and reduce the risk of immune evasion. This dual protection mechanism offers significant advantages in combating sudden animal outbreaks and providing comprehensive immune defense.

Future research should focus on the molecular mechanisms of trained immunity across different animal species. Ferreira et al. (39) highlighted that metabolic reprogramming and epigenetic modifications play pivotal roles in trained immunity, and interspecies differences in these areas may impact vaccine efficacy. Understanding these species-specific variations in trained immunity mechanisms will provide a theoretical basis for optimizing vaccine design. Additionally, developing safe, cost-effective, and scalable trained immunity inducers remains a critical task for the future. As Sánchez-Ramón et al. (27) described, trained immunity-based vaccines (TIbVs) activate broad-spectrum immune responses by recognizing microbial structures through pattern recognition receptors (PRRs), potentially offering broad protection against multiple pathogens.

Optimizing delivery methods and enhancing delivery efficiency are critical for ensuring that trained immunity inducers effectively reach target tissues and provide sustained effects. Nanocarrier systems, such as polylactic-co-glycolic acid (PLGA)-based nanoparticles and liposome-based platforms, offer promising solutions in veterinary vaccines. PLGA nanoparticles enable slow antigen release, promoting humoral immune memory and CD8+ T-cell responses (40), while liposomal vaccines have successfully delivered antigens against pathogens like *Salmonella enteritidis* (41). Priority should be given to developing trained immunity-enhanced vaccines for economically significant animal diseases, with demonstration projects providing models for broader applications. Collaborative efforts among research institutions, vaccine manufacturers, and regulatory agencies are essential to establish evaluation standards and streamline approval processes for these novel vaccines. With advancements in nanotechnology and production scalability, nanocarrier-based vaccines are poised to revolutionize veterinary vaccinology and significantly enhance disease control efforts.

## Data Availability

The Original contributions presented in the study are included in the article/supplementary material, further inquiries can be directed to the corresponding author.
